# The long-term effect of generic price competition on the Hungarian statin market

**DOI:** 10.1186/s12913-023-09431-6

**Published:** 2023-05-06

**Authors:** Balázs Répásy, Tibor Gazsó, Diána Elmer, Dalma Pónusz-Kovács, Fanni Luca Kajos, Tímea Csákvári, Bettina Kovács, Imre Boncz

**Affiliations:** 1 Faculty of Health Sciences, Institute for Health Insurance, University of Pécs, Vörösmarty U. 3, 7621 Pécs, Hungary; 2 National Laboratory for Human Reproduction, Ifjúság Útja 20, Pécs, 7624 Hungary

**Keywords:** Generic competition, Statins, Drug market, Blind bid, Margins

## Abstract

**Background:**

Generic competition is a vital health policy tool used in regulating the pharmaceutical market. Drug group HMG-CoA reductase (3-hydroxy-3-methyl-glutaryl-coenzyme-A reductase) inhibitors, widely known as “statins,” was the first drug group in Hungary in which generic prescriptions became mandatory. Our aim is to analyze the changes in the retail and wholesale margins through the generic competition regarding “statins”.

**Methods:**

Data was derived from the nationwide pharmaceutical database of the Hungarian National Health Insurance Fund Administration, the only health care financing agency in Hungary. We observed the turnover data regarding the HMG-CoA-reductase inhibitor “statins” from 2010 through 2019. As the drugs under review have a fixed price point in Hungary, we effectively calculated the margins.

**Results:**

In 2010, the consumer expenditure of statins was 30.7 billion HUF ($148 million), which decreased by 59%, to 12.5 billion HUF ($42.9 million) in 2019. In 2010, the annual health insurance reimbursement of statins was 23.7 billion HUF ($114 million), which underwent a 63% decrease to 8.6 billion HUF ($29.7 million) in 2019. In 2010, the DOT turnover was 287 million days, and it increased to above 346 million days for 2019, which reflects a 20% increase over the past nine years. The monthly retail margins decreased from 334 million HUF ($1.6 million), (January, 2010) to 176 million HUF ($0.61 million), (December, 2019). The monthly wholesale margins decreased from 96.3 million HUF ($0.46 million), (January, 2010) to 41.4 million HUF ($0.14 million), (December, 2019). The most significant downturn in margins was due to the introduction of the first two blind bids. The combined DOT turnover in reference to the examined 43 products consistently increased.

**Conclusions:**

The decline in retail and wholesale margin and in health insurance expenditures was largely due to a reduction in the consumer price of generic medicines. DOT turnover of statins also increased significantly.

## Background

Generics are products containing the same active ingredients and strengths as the original medicine, the equivalence of which are confirmed during a registry procedure. Once the original medication reaches its expiration date, due to patent protection legislation, the market becomes ripe regarding the appearance of an onslaught of generic products. In consideration of the appearance of generic and biosimilar products, the competition undergoes a steep increase resulting in a price breaking effect [1, 2]. Based on a cost-effectiveness study completed in Spain, in 2010, in reference to statins, the generic competition and reference price point has increased far beyond its cost-effectiveness [3]. The amount of price decrease is increasing with the number of generics [4–8]. Purchasers are interested in decreasing the cost of drugs, therefore, in many locations, including Hungary, they promote generic competition.

### A brief description of the Hungarian drug price reimbursement system

Within Hungary, there is one universal and compulsory social security system (National Health Insurance Fund Administration, NEAK) financing the entirety of the Hungarian public health care system. Due to the scarce resources of the country [9] and the demand for innovative medicine, it is required to decrease the cost of drugs, therefore, the national authority implements measures towards effectively increasing generic competition. It creates groups based on active ingredients and therapeutic areas (ATC code system), and within the groups the least expensive drug is awarded the highest level of health insurance reimbursement [10]. The drug awarded the highest health insurance support (i.e. "reference drug") the most prescribed alternative, therefore, the drug companies are interested in decreasing its costs.

Another way to reduce expenses was the introduction of "blind bid" processes introduced in October 2011. The matter of blind bid is the process in which NEAK publishes a call for competition among generic pharmaceutical entities, and requests these participating entities marketing the drugs to take an offer for the cost of their own product. However, when accepting an offer, the pharmaceutical entities do not know the cost offered by competitive entities, therefore the tendency is to drive down the introductory price point even more.

Within the process of a blind bid, mostly active ingredients are included which affects the vast number of inhabitants and is consumed by patients on a regular daily basis. The cost of many drugs decreased in bids one after the other, and this significantly decreased governmental social security support [11].

The detailed desciption of the structure of the Hungarian health care system [12–14] and financing features [15–19] can be found elsewhere.

Branded prescription or generic prescription is an important health policy issue in many countries [20–22]. In Austria, using state measures, the expenses regarding statins succeeded in decreasing the cost by 60% between 2001 and 2007 [23]. In accordance with a study completed in the Czech Republic, the use of statins increased from 2 to 96 DDT/TID (daily dose per thousand inhabitants) between 1997 and 2013, yet the expenses increased only 5.5 times. In the studied period, the prices of simvastatin and atorvastin decreased to 5% of its initial price [24]. The Chinese government implemented in 4 municipalities and 7 sub-provincial cities a volume-based drug procurement in 2019, referred to as "4 + 7" policy. In the policy pilot, price cut and cost-saving was observed in the antihypertensive drug category [25]. Notably, the benefits of price decrease were debated by each reference [26–29]. A Swedish study showed that adherence to statin refills was higher in patients who exposed regular substitution than in those who took the same medication throughout [30].

The price decrease has a distinct effect on patient burden throughout Hungary [31]. Regarding montelucast therapy, the accessibility was duly improved: the days of therapy was increased, and patient fees experienced a notable decrease. Simultaneously, the outflow of social security support also underwent a substantial decrease [32]. In the Hungarian drug market, supported drugs are accompanied with a fixed (authority) price point, therefore decreasing consumer prices result in decreasing margins throughout the retail and wholesale trade. Although the price decreasing effect regarding generic products are widely discussed in health-economic literature, there is considerably less available information regarding how the appearance of generics influences the characteristics of drug trade (wholesale and retail trade).

### Objectives

The objective of our analysis is to comprehensively understand how the generic price competition completed in the cholesterol decreasing (HMG-CoA reductase inhibitor) market of Hungary affected the DOT (Days of Therapy) turnover, the outflow of social security support, and the margin amount of retail and wholesale trade participants.

We selected statins in the primary focus of our study since this drug group requires significant social security reimbursement and affects a vast number of patients.

## Methods

### Data and methods of analysis

Our studies are based on the public drug trade database of NEAK. In the period between 2010 and 2019, trading and prices of the original product and generic drugs with statin active ingredient content was analyzed. In the analysis the following products included: Atorvastatin, fluvastatin, pravastatin, rosuvastatin and simvastatin. The number and changes of studied products are depicted in Table 1. In the table, we can see that the number of generic products decreased for fluvastatin, pravastatin and simvastatin. For these active substances, we can see a restricted market, but for rosuvastatin and atorvastatin, the generic market has expanded.

**Table 1:** Characteristics of medical products included in the analysis: number of generics and all generic alternatives in all strengths

		**2010**	**2011**	**2012**	**2013**	**2014**	**2015**	**2016**	**2017**	**2018**	**2019**
Atorvastatin	generic name	13	13	20	20	18	17	17	17	16	16
	strength	35	41	62	60	54	49	48	51	48	44
Fluvastatin	generic name	5	5	5	5	4	4	3	3	2	2
	strength	5	5	5	5	4	4	3	3	2	2
Pravastatin	generic name	2	1	1	1	1	1	1	1	0	0
	strength	6	3	3	3	3	3	3	3	0	0
Rosuvastatin	generic name	1	4	6	7	7	7	9	9	8	8
	strength	3	11	18	24	24	25	30	30	29	29
Simvastatin	generic name	12	12	13	12	10	11	10	9	9	7
	strength	37	36	39	35	31	30	27	26	26	21

Data included in the analysis originated from the drug trade database of the National Health Insurance Fund Administration presenting the pharmacy drug trade reimbursed by the Fund. In regard to our retrospective study, the necessary drug trade data were collected on a monthly base, and then these numbers were aggregated on an annual level. From the database DOT (Days of Therapy) turnover data, co-payment amount, and health insurance subsidy data were also collected, summarized in monthly and annual level.

Accordingly, the following indicators were used. DOT stands for "Days of Therapy" and refers to the total length of a medication treatment calculated based on the number of doses and duration of medication used during treatment [33]. The DDD is the assumed average maintenance dose per day for a drug used for its main indication in adults [34]. Co-payment is the gross amount paid by the patient for one box of medication. The patients’ co-payment amount is the total expenses of all patients. The health insurance subsidy is the gross amount of health insurance reimbursement paid by the health insurance fund. As the NEAK database contains only the supported drug turnover, we could not see the trade regarding products with a full cost or those without health insurance subsidy [35].

Next, the margin was calculated using the varied data. The authority margins in Hungary are banded, meaning, when increasing the value of the drug, costs increase in a decreasing rate (degressive) [36]. Using an algorithm, the retail and wholesale margin amount was calculated from trading data, which is the total profit margin divided between all wholesale and retail traders (pharmacies). Authority margins have undergone an increase by 1% since August 1, 2012.

## Results

**Fig. 1:**
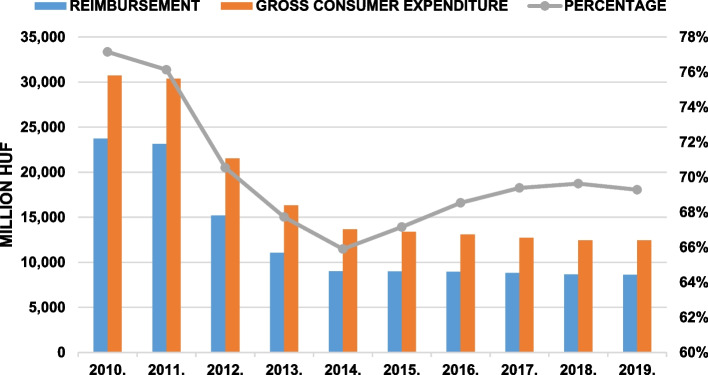
Gross consumer expenditure and the amount health insurance reimbursement of statin products

In Fig. 1, the relationship between consumer cost amount and health insurance subsidy is depicted annually. In 2010, the gross consumer expenditure of statins was 30.7 billion HUF ($148 million), which decreased by 59%, to 12.5 billion HUF ($42.9 million) in 2019. In 2010, the health insurance reimbursement of statins was 23.7 billion HUF ($114 million), which underwent a 63% decrease to 8.6 billion HUF ($29.7 million) in 2019. Consequently, the average measure in health insurance subsidy decreased from 77 to 69%.

**Fig. 2:**
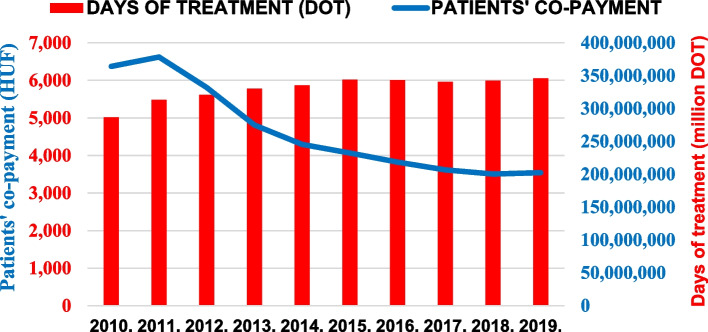
Number of days of therapy (DOT) and amount of patient’s co-payment

Figure 2 illustrates the change in DOT turnover and the co-payment payed by patients. In 2010, the DOT turnover was 287 million days, and it increased to above 346 million days for 2019, which reflects a 20% increase over the past nine years. Nevertheless, the co-payment underwent a decrease from 6.4 billion HUF ($30.6 million) (2010) to 3.5 billion HUF ($12.2 million) (2019), which represents a substantial 45% fall.

**Fig. 3:**
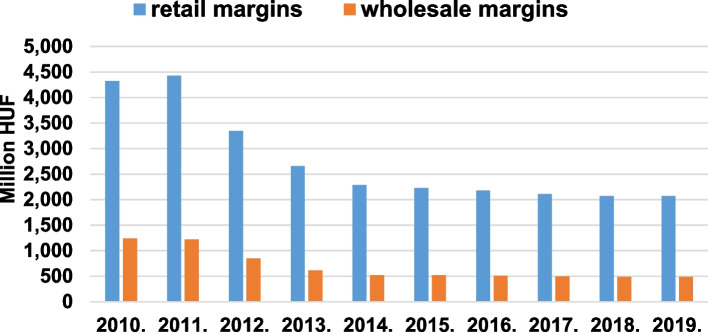
Annual retail and wholesale margin of products of statin market

Figure 3 annotates the annual margin amount decrease in the retail and wholesale sector. In 2011, the peak regarding the retail margin amount was 4.4 billion HUF ($22 million), and it experienced a 52% downturn to 2.1 billion HUF ($7.1 million), in 2010. The wholesale margin amount decreased from 1.2 billion HUF ($5.97 million), (2010) to 0.5 billion HUF ($1.67 million), (2019), resulting in a 58% loss. The small increase of margins in 2011 was due to the increasing DOT.

**Fig. 4:**
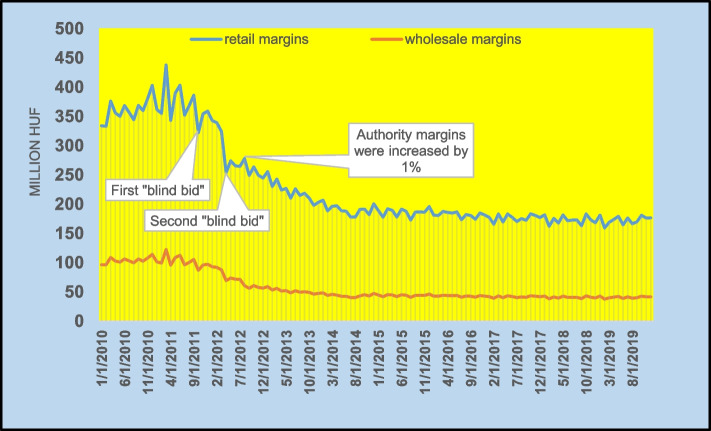
Monthly retail and wholesale margin of products of statin market

Figure 4 illustrates the monthly change in the retail and wholesale margin amounts. The fall in October 2011 occurred immediately following the first blind bid. The retail and wholesale margin amount underwent a decrease from 386 million HUF ($1.92 million) to 322 million HUF ($1.6 million) (by 15%), and from 105 million HUF ($0.52 million) to 87 million HUF ($0.43 million) (by 17%) in one month, respectively. The second blind bid in April 2012, resulted in a higher margin amount fall. The retail and wholesale margin amount were decreased from 324 million HUF ($1.44) to 253 million HUF ($1.12 million) (by 27%), and from 88 million HUF ($0.39 million) to 69 million HUF ($0.31 million) (by 22%) in one month, respectively. Blind bids remained during a fixing process held twice a year, however, experienced diminished relevance on the statin market. Margins were increased by 1% from August 1, 2012.

## Discussion

In our manuscript, we analysed the DOT turnover, the outflow of social security support, patient’s co-payment and the change in the retail and wholesale margins through the generic competition of the HMG-CoA-reductase inhibitor “statins.”

Following the decline in consumer price, the co-payment paid by patients, the annual health insurance subsidy and both the retail and wholesale margins underwent a significant decrease. Nevertheless, DOT trade experienced an increase, in which the accessibility regarding the product throughout Hungary was significantly improved.

In South Korea, from 2006 through 2010, the cost of cholesterol reducers underwent a decrease due to three state implemented measures following one after the other. In published papers, the decrease in consumer costs leads to the potential expansion of the generic market, and this paradoxically increases expenses. The supportive reasoning implies the trend is the result of the exploding DOT trade, and the number of patients treated with atorvastatin doubled monthly [29]. In the rapidly increasing generic marketplace, the decrease of prescribed dosages could not result in an expense decrease [37].

In Scotland, NHS initiated a significant generic program with unlimited to preferential prescriptions based on active ingredients and generic products instead of originals. As a result of this program, the amount of social security expenses paid for cholesterol decreasing products was decreased by 50%, however, in parallel, the use of drugs underwent a substantial 412% increase. [38]

Drug policy restrictions do not always have an effect on increasing use of statins, especially if they increase the out-of-pocket expenses. According to a study conducted in Italy between May 2001 and December 2007, drug policy interventions had a negative effect on the growth trend of statin consumption [39].

To improve patients' access to new drugs under uncertainties, South Korea have adopted risk sharing arrangements in 2013. This intervention provided a dual success of containing pharmaceutical expenditure and improving access to expensive drugs [40].

Based on published European research, the cooperation regarding pharmacists is highly important towards expanding the generic market. According to a recently published European report, fixed margin should be introduced instead of price dependent margin, and then marketing the generic drug instead of the original would not lead to any loss regarding the business of pharmacies [41].

In a study in South Africa, between January 2012 and December 2015, the turnover, cost and drug expenditures of candesartan and rosuvastatin were studied following the patent expiration. The turnover of candesartan and rosuvastatin underwent an annual decrease by 7%, and 5% average, respectively. Expenditures decreased by 34.6% and 20.9% for candesartan and rosuvastatin, respectively. The total savings was 54.8% and 31.9% for candesartan and rosuvastatin, respectively. The appearance of generic drugs and the introduction of generic reference price points did not affect the use of drugs, however, the trend resulted in a decrease regarding the cost and expenditure of both candesartan and rosuvastatin [42].

In an international study, published in 2018, the statin market of Australia, New Zealand, the Republic of Korea and Singapore was compared, from 2006 through 2015. Prior to marketing generic atorvastatin, the lowest price ($0.10/DDD) was in New Zealand, and the highest price ($2.89/DDD) was in the Republic of Korea. However, the cost regarding DDD experienced a notable decrease with the appearance of generics in all countries, with the exception of New Zealand, in which the costs were already considerably lower. The highest decrease was seen in Singapore (46% in year 1). In consideration of the fourth year, following the appearance of generic forms of medication, the price in each country underwent a 46–80% decrease; however, among these countries, high differences relative to opening price was observed. The tendering or bidding system in New Zealand and the use of preferred drugs resulted in low atorvastatin price even prior to the expiration of its patent. In three other countries, the pricing policy effectively decreased the cost of atorvastatin: in 4 years following the appearance of generics it underwent a substantial 46–80% decrease [43].

In Denmark, from 1996 through 2012, the prescription of simvastatin (the generic version first introduced in 2002) experienced an increase from 1.2 DDD/1000 inhabitants (55% of total statin use) to 86 DDD/1000 inhabitants (74% of total statin use). Afterwards, in 2015, the use underwent a decease to 72 DDD/1000 inhabitants (53% of total statin use). The use of atorvastatin was slowly increased from its first appearance in the marketplace, in 1997, up through 2012, when its generic version was introduced. After 2012, a sudden, striking increase was observed, and in 2015, 55 DDD was reached (40% of total statin use). Since its introduction in 2003, the use of rosuvastatin was continuously increased from 0.2 DDD/1000 inhabitants (1% of total statin use) to 9.5 DDD/1000 inhabitants in 2015 (7% of total statin use). The common use of remaining statins (lovastatin, pravastatin, fluvastatin, and cerivastatin) reached 3.2 DDD/1000 inhabitants in 2002 (21% of total statin use), and then in 2015, it fell to 1.1 DDD/1000 inhabitants (< 1% of total statin use) [44].

Tesar and colleagues investigated the impact of selected legislative initiatives and their implementation for off-patent medicinal products in Slovakia compared with the rest of the Visegrád Group (V4 countries). They found that the sales of generic drugs in Poland fell from 40.4% in 2015 to 35.0% in 2020, from 26.2 to 22.1% in Hungary, and from 29.6 to 20.4% in Czechia. [45] Simoens analyzed the Polish generic market and concluded that Poland needs to consider moving away from competition by discount to competition by price [46].

Tkachova et al. analysed the affordability of statins therapy in Ukraine and Bulgaria. They found that the most affordable treatment is a generic atorvastatin in Bulgaria and generic rosuvastatin in Ukraine. [47] Naumovska and colleagues evaluated the market share and utilization trends of statins in the Republic of Macedonia. They concluded that the statin use increased from 42.347 DDD/ thousand inhabitants per day (TID) in 2013 to 71.697 DDD/TID in 2016, although it is still lower in comparison to other European Union (EU) countries (1.1–2.5-fold) [48]. Makarevicius et al. analysed the trends in statin utilization in Lithuania. Statin use increased from 8.28 DDD/TID in 2010 to 96.06 DDD/TID in 2021. [49].

The most important aspect regarding the Hungarian health insurance fund’s (NEAK) generic program is that generic companies can get a market authorization for their drugs by using a simplified registration procedure, in which only bioequivalence shall be proven. As a result, after the patent expiration, an increase in generic forms of medication is likely to be seen in the Hungarian pharmaceutical market. Generic medication will then be in stiff competition with one another, and the entity at the lowest price will gain a market advantage.

To effectively market and promote a generic program throughout Hungary, the prescription based on active ingredients was introduced on April 1, 2012, regarding statin active ingredients groups [50]. In the study period, due to the decreased drug prices, the turnover of pharmacies was decreased to the point, when new governmental regulations had to be implemented to maintain the sustainability of the drug supply. From August 1, 2012, both the retail and wholesale margins were increased by introducing a generic promoting system and service fees received by pharmacies based on the prescription turnover. [51], Among the limitations of the study, it should be mentioned that the number of generic products decreased during the examination period, and that we did not have insight into the small and wholesale turnover of medicines that do not have governmental support. These have an impact on the pharmaceutical turnover and the supply of medicines to patients.

## Conclusions

In the examined period, the generic program (the blind bidding) reduced drug prices, drug expenditures and increased DOT turnover in Hungary. The decline in retail and wholesale was largely due to a reduction in the consumer price of medicine. The loss could not be balanced even by the increase in DOT turnover. The outflow of health insurance reimbursement has also decreased. The pharmaceutical policy intervention was beneficial from the point of view of the patients and the health insurance fund but had unfavourable effects from the point of view of the pharmaceutical trade.

## Data Availability

Data was derived from the nationwide pharmaceutical database of the Hungarian National Health Insurance Fund Administration: http://www.neak.gov.hu/felso_menu/szakmai_oldalak/publikus_forgalmi_adatok/gyogyszer_forgalmi_adatok
